# Opportunities to tackle antibiotic resistance development in the aquatic environment through the Water Framework Directive

**DOI:** 10.1007/s13280-022-01828-7

**Published:** 2023-02-01

**Authors:** Marlene Ågerstrand, Henrik Josefsson, Ann-Sofie Wernersson, D. G. Joakim Larsson

**Affiliations:** 1 Department of Environmental Science, Stockholm University, Svante Arrhenius väg 8, 106 91, Stockholm, Sweden; 2 Department of Business Studies, Commercial Law Unit, Uppsala University, Kyrkogårdsgatan 10, 751 20 Uppsala, Sweden; 3 Department for Management of Contaminated Sites, Swedish Geotechnical Institute, Hugo Grauers gata 5 B, 41296 Göteborg, Sweden; 4 Department of Infectious Diseases, Institute of Biomedicine, University of Gothenburg, Guldhedsgatan 10A, 413 46 Gothenburg, Sweden; 5 Centre for Antibiotic Resistance Research, University of Gothenburg, Gothenburg, Sweden

**Keywords:** Antimicrobial resistance, AMR, Chemical regulation, EQS, Quality standards, Water Framework Directive

## Abstract

Antibiotics are critical components of modern health care. Protecting their efficacy through managing the rise in antibiotic resistance is therefore a global concern. It is not known to what extent environmental pollution from antibiotics contributes to the development of resistance, but encountered concentrations are frequently above concentrations predicted to select for resistance. Hence, measures are needed to manage risks. Here, we analyse if the indirect health risks from antibiotics in the aquatic environment can be considered in the context of the EU Water Framework Directive and the setting of environmental quality standards (EQS). By scrutinising current legislation, we conclude that it is possible to take the indirect health risks from antimicrobial resistance into account when deriving EQS for substances with antibiotic activity. We base this on the following conclusions: (1) human health concerns can be the main driver when setting an EQS, (2) an EQS can be based on data not specified in the guidance document, and (3) there are no restrictions against establishing EQS using data on antimicrobial resistance properties. In addition, since antimicrobial resistance travel across borders, we see strong reasons to prioritise setting these EQS on the EU level over the national level. Even though there is no agreed-upon method for how to develop EQS protective against resistance selection, there are several suggestions available in the literature and a couple of examples of regulatory initiatives. Also, addressing antimicrobial resistance through the Water Framework Directive can act as a driving force for other applicable legislation where such risks are not considered. We end by providing a set of recommendations for the European Commission and the Members States' future work on addressing aquatic pollution and antimicrobial resistance.

## Introduction

Antibiotics are critical components of modern health care and veterinary medicine for treating and preventing bacterial infectious diseases. Infections caused by antibiotic-resistant bacteria have become among the leading causes of death worldwide, ahead of both HIV/AIDS and malaria (Murray et al. 2022). Protecting the efficacy of antibiotics through managing the rise in antibiotic resistance has become a global concern. Important actions include initiatives to reduce misuse and overuse of antibiotics in both humans and domestic animals, phase out the use of antibiotics as animal growth promoters, and reduce transmission through improved sanitation and enhanced hygiene, both in health care and in animal farming operations. However, of rising concern is also the contamination from antibiotics and other antimicrobial substances of the environment, primarily linked to excretion from humans (sewage), domestic animals (including fisheries), and emissions from the manufacturing of antibiotics (Larsson and Flach 2022; Bengtsson-Palme et al. 2018; Kümmerer 2009a, b).

It is a complex task to fully understand and be able to assess the (indirect) human health risks associated with antibiotic pollution of the environment (Ashbolt et al. 2013; Larsson and Flach 2022). It is believed that many of the resistance factors we face in the clinic today originate from bacteria present in the environment (Ebmeyer et al. 2021). While selection pressure from antibiotics is recognised as a critical, and probably the most critical, factor in the evolution of resistance in pathogens, it is not known to what extent environmental pollution from antibiotics contributes to this evolution. Even less is known about the implications of other antimicrobial substances such as antibacterial biocides and certain metals also being present in the environment. However, encountered concentrations of antibiotics in industrially polluted surface waters are frequently above the minimal inhibitory concentration (MIC) for wild-type bacteria, i.e. concentrations that inevitably select for resistant genotypes under at least some circumstances (Bengtsson-Palme and Larsson 2016). Untreated hospital wastewater and municipal wastewater may also select for multi-resistant bacteria, as recently shown (Kraupner et al. 2021). There is also widespread concern about the selection of resistance at the lower levels of antibiotics regularly encountered in waterways contaminated by such waste (Wilkinson et al. 2022).

The risk of antibiotics or other antimicrobial agents contributing to the evolution of antimicrobial resistance (AMR) in the environment (water, soil, etc.) is to our knowledge currently not incorporated in any environmental legislation in the EU. Whether this is due to simply overlooking this aspect, lack of knowledge or sufficient evidence of the significance of the environmental pathways, lack of methodology, lack of a suitable regulatory framework to deal with the problem, or a combination of several factors, is not clear. Nevertheless, the European Commission has acknowledged that AMR via the environment is a well-documented health risk; for example, diseases in humans and animals are established due to new resistant microorganisms found in both soil and water (European Commission 2019a).

The European Commission, UNEP, and WHO, as well as individual countries, have developed action plans and/or guidance for policymakers addressing the environmental contribution to the development of AMR (European Commission 2017; WHO 2015; UNEP 2022; Government of India 2017; Government Offices of Sweden 2016). The European Commission in its policy documents position itself and the EU at the forefront of combating AMR. Declining authorisation of new antibiotic products for human use due to risks with resistance development through environmental emission seems unlikely, given the increasing global need to find new treatments. Other types of mitigations than declining market approval are therefore warranted if there is a concern for environmental selection. Harmonised environmental monitoring of antimicrobials and microorganisms resistant to antibiotics and new methods for evaluating risks to human and animal health are two of the priorities (European Commission 2017, 2019b). In its strategic approach to pharmaceuticals in the environment, the Commission considers the feasibility of monitoring AMR regarding microorganisms and antimicrobial resistance genes under the Water Framework Directive (2000/60/EC) (European Commission 2020). This leads us to delimit this paper to whether the Water Framework Directive (and the accompanying Environmental Quality Standards Directive, 2008/105/EG) could become a driver in reducing AMR in the environment.

### Water Framework Directive

The Water Framework Directive is a key directive when it comes to regulating the quality of European ground and surface water. It sets out the ambitions for the water environment with a set of objectives aiming at reducing human impact. The chemical surface water status is classified based on the concentrations of a predefined set of substances and EU-wide Environmental Quality Standards (EQS), i.e. the concentration of a particular pollutant or group of pollutants in water, sediment, or biota which should not be exceeded to protect human health and the environment (Article 2(35)). As of today, there are 45 priority substances but none of them are antibiotics or other pharmaceutical substances. However, there are antibiotics and antifungals on the watch list and three antibiotics among the priority substance candidates (azithromycin, clarithromycin, and erythromycin) (Table 1).

**Table 1:** Antimicrobial pharmaceuticals included in the Water Framework Directive watch list (WL) and/or current priority substance candidates and their preliminary evaluation criteria from 2021 along with PNEC-MIC values suggested by the AMR Industry Alliance (2018) and established EQS in individual European countries.

Substance	WL-PNEC and/or preliminary EQS dossier	PNEC-MIC	National EQS
Amoxicillin	WL-PNEC 0.078 µg/L (Loos et al 2018)	0.25 µg/L	Denmark (Danish Environmental Protection Agency 2006a): AA-EQS 0.078 µg/l in freshwater and marine MAC-EQS 0.37 µg/l
Azithromycin	WL-PNEC 0.09 µg/L (Carvalho et al 2015) Preliminary EQS dossier: AA-EQS 0.019 µg/L (freshwater) and 0.0019 µg/L (marine) MAC-EQS 0.019 µg/L (freshwater) and 0.018 µg/L (marine) QS_secpois_* 0.009 µg/L (limnic) QS_sediment_ 16.92 µg/kg dw (limnic) and 1.692 µg/kg dw (marine)**	0.25 µg/L	Switzerland (814.201 Water Protection Ordinance (WPO)): AA-EQS 0.019 µg/L MAC-EQS 0.18 µg/L***
Ciprofloxacin	WL-PNEC 0.089 µg/L (Carvalho et al 2015)	0.06 µg/L	Sweden (Swedish Agency for Marine and Water Management 2019): MAC-EQS 0.1 µg/L
Clarithromycin	WL-PNEC 0.13 µg/L (Carvalho et al 2015) Preliminary EQS dossier: AA-EQS 0.13 µg/L (freshwater) and 0.013 µg/L (marine) MAC-EQS 0.13 µg/L (freshwater) and 0.013 (marine) QS_secpois_* 5.82 µg/L QS_dw,hh_ 9.8 µg/L QS_hh, seafood_* 2,02 µg/L	0.25 µg/L	Switzerland (814.201 Water Protection Ordinance (WPO)): AA-EQS 0.12 µg/L MAC-EQS 0.19 µg/L***
Clindamycin	WL-PNEC 0.044 µg/L (Gomez Cortes et al. 2022)	1 µg/L	
Clotrimazole	WL-PNEC 0.02–1 µg/L (Cortez et al., 2020) Sediment 31.6 ug/kg wwt	-	
Erythromycin	WL-PNEC 0.2 µg/L (Carvalho et al 2015) *Preliminary EQS dossier*: AA-EQS 0.5 µg/L (freshwater) and 0.05 µg/L (marine) MAC-EQS 0.523 µg/L (freshwater) and 0.0523 (marine) QS_secpois_* 0.102 µg/L QS_dw,hh_ 7000 µg/L QS_hh, seafood_* 106 µg/L QS_sediment_ 6.02 µg/kg dw (limnic) and 0.6 µg/kg dw (marine)**	1.0 µg/L	
Fluconazole	WL-PNEC 0.25-9.46 µg/L (Cortes et al. 2020) Lowest refers to PNEC-MIC	0.25 µg/L	
Miconazole	WL-PNEC 0.2–0.044 µg/L (Cortes et al. 2020)	-	
Ofloxacin	WL-PNEC 0.026 µg/L (Gomez Cortes et al. 2022)	0.5 µg/L	
Sulfamethoxazole	WL-PNEC 0.1–0.4 µg/L (Cortes et al. 2020)	16 µg/L	
Trimethoprim	WL-PNEC 0.5–120 µg/L (Cortes et al. 2020) Lowest refers to PNEC-MIC	0.5 µg/L	Denmark (Danish Environmental Protection Agency 2006b): AA-EQS 100 µg/l in freshwater and 10 µg/l in marine MAC-EQS 160 µg/l

*dw* dry weight, *wwt* wet weight

^*^For comparison only, QS values are here expressed for water concentrations, in the dossiers derived by recalculation from biota (prey or fishery products). **Based on equilibrium partitioning calculations (sediment ecotoxicity data are absent). ***The value should be compared to the concentration determined over 2 weeks, this differs from the EU regulation where the annual average concentration is used

On a national level, ecological status is classified based on a combination of biological, hydromorphological, chemical, and physicochemical quality elements (Josefsson 2015). Member States identify and set national EQS for additional substances, referred to as river basin-specific pollutants. These EQS are used to distinguish between good and moderate ecological status. To our knowledge, national EQS for antibiotics are so far included in Sweden (ciprofloxacin), Switzerland (clarithromycin and azithromycin), and Denmark (amoxicillin and trimethoprim, and planned for azithromycin, ciprofloxacin, clarithromycin, and erythromycin) (Table 1).

The procedure for the setting of EQS is described in detail in the Common Implementation Strategy (CIS guidance no 27 (European Commission 2018). The EQS is based on the most stringent value of several Quality Standards (QSs) that are set for the following protection goals: pelagic organisms (QS_fw_, _eco_ and QS_sw, eco_ for freshwater and saltwater environment, respectively), benthic organisms (QS_sediment_), predators (QS_biota_, _secpois_), and humans through exposure to drinking water (QS_dw,hh_) and consumption of fishery products (QS_biota,hh food_). AMR is not mentioned in the guidance but in the chapter on extrapolation it is stated that “*uncertainty may be increased if data for sensitive taxa are missing when dealing with substances with a specific mode of action like insecticides, herbicides, or antibiotics. Under these circumstances, an assessment factor larger than the default may be warranted.*”

The European Commission has established a watch list of substances for which union-wide monitoring data are gathered to support future prioritisation exercises. Up until the 3^rd^ watch list, only PNECs (Predicted No-Effect Concentrations, similar to EQS) addressing ecotoxicity to aquatic organisms were used for the prioritisation of antibiotics (erythromycin, clarithromycin, azithromycin, ciprofloxacin, amoxicillin, sulfamethoxazole, and trimethoprim) in the EU (Carvalho et al. 2015; Loos et al. 2018; Cortes et al. 2020), even though PNEC for resistance selection were mentioned for sulfamethoxazole and trimethoprim (Cortes et al. 2020). In the three documents, the need for information on exposure to antibiotics because of the increasing concern regarding AMR is mentioned among the reasons to consider them for inclusion in the watch list. It is also stated regarding antibiotic substances that “*apart from being toxic they may contribute to the spread and persistence of antimicrobial resistance (AMR). The selection of Anti-Microbial pharmaceuticals (antibiotics and antifungal agents) is also in line with the European One Health Action Plan against antimicrobial resistance (COM/2017/0339 final*)”. In the 4^th^ watch list, the two antibiotics clindamycin and ofloxacin were included and the European Commission is again referring to the use of the watch list to improve knowledge of the occurrence and spread of antimicrobials in the environment (European Commission 2022a). This is despite the described lack of standardised approaches for assessing the risks (Kraupner et al. 2021; Larsson and Flach 2022).

### Aim

This study aims to analyse if the indirect health risks from antibiotics being present in the aquatic environment can be considered in the context of the Water Framework Directive. Our interest was to investigate if it would be possible, from a regulatory perspective, to take the indirect health risks through AMR into account when deriving EQS for substances with antibacterial activity, such as antibiotics.

More specifically, the following research questions were addressed:

- Can human health concerns be the main concern when setting an EQS?
- Can data not specified in the EQS guidance document be used when setting an EQS?
- Can data on risks for the selection of AMR be used when setting an EQS?
- Should EQS based on selection risks for AMR be set on a national or EU level?
- How can changes in the implementation of the Water Framework Directive and EQS setting affect other regulations in this context?

## Materials and methods

The legal possibilities were investigated using a method where the legal material, preparatory works, Directives, Case law, Commission Decisions, Soft Legal Material (such as CIS 27), and Doctrine were analysed based on the theological interpretation model developed by the EU Court of Justice (Lenaerts and Gutiérrez-Fons 2014; Fennelly 1996). The theological model aims to uncover the purpose of legislative acts and, for example, if this act applies to a certain question. Central to the theological methods are the objectives and purposes of the legislation and how these are constructed through several definitions. Also important is if the legislation is a minimum directive or results in the harmonisation of national legislation, and how the legislation is aimed towards promoting the achievement of the objectives pursued by the EU Treaty. In this context the method aims to answer if there is legal support in the Water Framework Directive to (1) take resistance selection into account and (2) whether the development of an EQS set to protect against resistance selection would fit into the current legal text. Thus, the method, in this case, results in an interpretational process that inquires where the limits of the Water Framework Directive objective/s and, more specifically the EQS “construction” are.

## Results

### The human health concern is one driver

The Water Framework Directive was constructed due to a need for a community water policy covering ecological quality and to develop existing water quality laws mainly focused on pollution (European Commission 1997). The Water Framework Directive, therefore, aims at maintaining and improving the quality of the aquatic environment in the EU (Article 1). The Water Framework Directive achieves this aim for polluting substances by specifying that a progressive reduction of emissions of hazardous substances to water must be achieved jointly by all Member States (Recital 22). The Member States should “*adopt measures to eliminate pollution of surface water by the priority substances and progressively to reduce pollution by other substances which would otherwise prevent Member States from achieving the objectives for the bodies of surface water*” (Recital 45). The Water Framework Directive is to a large extent based on the idea that when substances pollute the water environment this results in negative consequences for the aquatic ecosystem and humans (Article 2 (21, 33, 35), 16 (2)(b)).

When drafting the Water Framework Directive, substances that are primarily of concern for human health seem not to have been the main focus (European Commission 1997). Still, threats to human health are included in the Environmental Quality Standards Directive (recital 1) and for some substances, such as PFOS (Perfluorooctanesulfonic acid) and mercury, the derived EQS is driven by the concern for human health and/or secondary poisoning of mammals and birds feeding on fish, although also protecting against effects that could be observed in the aquatic environment itself (European Commission 2011, 2005). For river basin-specific pollutants, it is less clear from the Water Framework Directive that human health should be considered when setting the EQS. Still, this is a matter of Member State discretion, as this is not an area that is harmonised. Nevertheless, the same technical guidance (European Commission 2018) is used for both priority substances and river basin-specific substances. Thus, although the river basin-specific pollutants are considered in setting the ecological status, health risks can also be considered when deriving a national EQS. Therefore, we can conclude that there is no formal obstacle to establishing an EQS for a substance on either EU or national level, for which human health concern is the main driver.

### The Water Framework Directive is a minimum directive

The Water Framework Directive stipulates the results to be achieved, but leaves to the national authorities the choice of form and methods, meaning that the Member States can choose to go further (Article 288 The treaty on the functioning of the European Union). Through the Common Implementation Strategy (CIS) a large number of guidance documents and other technical reports have been published to support the implementation of the Water Framework Directive. These guidance documents are not legally binding and it is not known to us to what extent more than required is done by the individual Member States. For the use of EQS for resistance selection on the EU level, from a legal perspective, it must be acceptable according to both the Water Framework Directive and Environmental Quality Standards Directive. We note that EQS for priority substances should be developed to ensure that the aquatic environment and human health are adequately protected (see above, and recitals 13, 27 and 32 of the Environmental Quality Standards Directive). The regulation at the EU level is in the interest of effective regulation of surface water protection to set the appropriate set of EQS and leave it to the Member States to lay rules for remaining pollutants at the national level (recital 11 EQS-Directive). For river basin-specific pollutants, the Water Framework being a minimum directive, Member States can move beyond possible limitations stipulated in Annex V.1.2.6 and take also other protection goals into account as long as the national EQS is based on the most stringent value. Therefore, we can conclude that there is no formal obstacle for the European Commission, or a Member State, to establish an EQS for a substance based on additional data not specified in the EQS guidance document (CIS 27) as long as it is still in line with the ambition of the Water Framework Directive or left to Member State discretion.

### Including risks for driving AMR is possible

In the Water Framework Directive, “pollutant” means any substance liable to cause pollution, in particular those listed in Annex VIII (such as biocides and plant protection products, but also substances contributing to eutrophication, have an unfavourable influence on oxygen balance, as well as materials in suspension [Article 2(31)]). “Pollution” on the other hand is defined as *“the direct or indirect introduction, as a result of human activity, of substances or heat into the air, water or land which may be harmful to human health or the quality of aquatic ecosystems or terrestrial ecosystems directly depending on aquatic ecosystems, which result in damage to material property, or which impair or interfere with amenities and other legitimate uses of the environment”* [Article 2(33)]. “EQS” is defined as the concentration of a particular pollutant or group of pollutants in water, sediment, or biota which should not be exceeded to protect human health and the environment [Article 2(35)].

There is a constant discussion and evolvement of what is considered a suitable endpoint for inclusion in regulatory assessments for chemicals. So far, the discussion has focused on the different types of toxicological effects, such as behaviour (Ågerstrand et al. 2020), and histopathological changes (Maack et al. 2022). Besides the direct toxic effect of antibiotics itself, resulting in potential effects on microbial community structure and ecological functions, such as nutrient regeneration, organic matter mineralisation, and pollutant degradation, environmental antibiotic resistance as such does not constitute a *direct* risk for aquatic ecosystems. The human health concern with antibiotics in the environment is not primarily that human exposure to environmental antibiotic residues should lead to direct toxicity or even drive resistance within the human microbiota. It is rather that it would lead to selection of resistant microorganisms within the environment, contributing to the risks for evolution and subsequent spread of resistance, which down the road may have major public health consequences (Larsson and Flach 2022). Therefore, antimicrobials in the environment pose an *indirect* risk to human and animal health in that sense, it differs from the direct toxicological effects of most other environmental pollutants. Knowing this, it can be discussed if the selection of AMR fulfils the requirement of the second part of the definition of “pollution”, which the definition of EQS is based on, i.e. “*may be harmful to human health*”. Although not written explicitly in the definition of pollution one could argue that “*harmful to human health*” refers to substances being *directly* harmful (toxic) to humans. However, a wider interpretation of this definition that looks at the purpose of the Water Framework Directive could argue that also pollution from a substance that may be *indirectly* harmful to human health is of concern for the Water Framework Directive. Furthermore, in Article 2(29) “hazardous substances” are defined as substances or groups of substances that are “*toxic, persistent, and liable to bioaccumulate, and other substances or groups of substances which give rise to an equivalent level of concern*”. “Toxicity” typically refers to carcinogenic, mutagenic and reprotoxic (CMR) properties, while “equivalent level of concern” has had a more flexible definition. Within the REACH regulation, the “equivalent level of concern” has included endocrine disruption, sensitisation, as well as immunotoxic and neurotoxic properties (European Chemicals Agency, n.d.; Quiros Pesudo and Aschberger 2015). To our knowledge, risks for driving AMR have so far not been suggested as an “equivalent level of concern” in any EU regulations but considering that the definition is adaptive we conclude that there is no formal obstacle to establishing an EU-wide or national EQS for a substance based on the selection of AMR.

### EQS preferably to be set on the EU level

In practice, there are two possible venues for EQS for resistance selection, either on the EU level (EQS for priority substance) or the Member State level (EQS for river basin-specific pollutants). The inclusion of antibiotics and other antimicrobial substances in the Environmental Quality Standards Directive and the development of EQS on the EU level would be beneficial as this stipulates that the Member States need to monitor these substances in water bodies and regulate the most important polluters, if and where concentrations exceed the EQS. Furthermore, the emissions would need to be reported and kept at a minimum. The alternative for this is to establish EQS on a national level. So far only the Swedish EQS for ciprofloxacin has been established to also protect against AMR (Sahlin et al. 2018), further described below. The Member States are obliged to identify pollutants other than those on the priority list discharged in significant quantities into water bodies as river basin-specific pollutants and to set and meet EQS for them. However, in a fitness check of the Water Framework Directive and the Floods Directive, it was concluded that the “*second-cycle RBMPs* [i.e. River basin management plans] *show a larger variability than can be explained by location-specific conditions”* and that the Member States “*do not consistently identify all relevant substances as river basin specific pollutants, or do not report the same extent of failure to meet the EQS for the relevant river basin specific pollutants, even when a substance is present at the same concentration. This is an instance where the flexibility left to the Member States leads to sub-optimal results”* (European Commission 2019b). A key message in this context was that because the legal deadline for meeting the environmental quality standards for river basin-specific pollutants cannot be adapted, it “*does not encourage Member States to add substances to their lists, even though it is important to regularly update the lists of substances based on up-to-date knowledge”.*

This, and the fact that the consequences of AMR are of an EU-wide (and global) concern (see below), speaks for including EQS for antimicrobials and their potential to select for AMR under chemical status and not leaving it to each Member State to decide whether to set and implement the EQS when assessing ecological status. Furthermore, the monitoring requirements for river basin-specific pollutants are lower, there is no legal obligation to report emission data as part of the inventory, and it is not necessary to keep the emissions at a minimum, only to make sure the EQS is not exceeded.

To include a substance in the Environmental Quality Standards Directive and establish an EU-wide EQS requires that the substance is an EU-wide problem that is shared between the Member States (European Commission 2018). The scientific literature and actors such as the European Commission seem clear that AMR is a common and most serious human health concern (European Commission 2017). In addition, pharmaceutical pollution, including the release of antibiotics and the associated risks for promoting resistance, is a joint concern, as reflected very clearly in the EU's strategic approach to pharmaceuticals in the environment (European Commission 2019a). The emergence of new forms of resistance in pathogens is a relatively rare, still reoccurring event. Resistance genes are mobilised and transferred to pathogens, one after the other, leading to greater and greater difficulties in finding efficient cures. These “emergence events” are irreversible in the sense that once a resistance gene has established itself in a given pathogen species, it cannot be eradicated (Larsson and Flach 2022). A successful resistance genotype that first appears in bacteria at one location could rapidly spread to other locations. Once a resistant pathogen has established itself in a host population (humans or animals), within a short time frame (weeks, years, depending on transmission opportunities and travel patterns), the resistant pathogen can easily spread to and infect hosts living in other states and continents (Johnson and Woodford 2013).

Leaving the risks for selection of AMR to be solved by the Member States as river basin-specific pollutants, under ecological status, could result in some Member States not acting accordingly, which then can result in accelerating the development and spread of AMR within the EU and worldwide. Although many studies have tried to estimate the overall costs associated with AMR (Dadgostar 2019), it is very difficult to define costs specifically related to environmental pollution with antimicrobials. Not least this is because it is difficult to quantify to what extent antibiotic selection pressures in the environment are involved in the emergence and evolution of resistance (Ebmeyer et al 2021; Larsson and Flach 2022). The view expressed not only by the EU (European Commission 2019a) but also by industry (AMR Industry Alliance. 2018) indicates that even with such uncertainties, too much is at stake for not acting on antimicrobial pollution. However, the process of establishing EQS for antimicrobial residues and risk for selection of AMR under the EQS directive probably takes a longer time than establishing river basin-specific pollutants nationally and as we could conclude, there are no formal obstacles to including AMR-driven EQSs in the assessment of ecological status. Current ambitions from some Member States, such as Sweden, to establish national EQSs that also protect against AMR should therefore continue and be encouraged as long as the European Commission has not decided to move forward with an EU-wide EQS on AMR for the substance.

### The Water Framework Directive can act as a driving force

The Water Framework Directive has a central role within EU water policy. Thus, setting EQS that take AMR into account would have implications also in other regulatory contexts. It is, for example, clearly stated in the Urban Waste Water Directive (91/271/EE, Annex I B4) on the requirements regarding discharge to receiving waters, that “*More stringent requirements than those shown in Table 1 and/or Table 2 shall be applied where required to ensure that the receiving waters satisfy any other relevant Directives*”. It refers indeed to requirements according to other directives, in this case (referring to “receiving waters”), most likely primarily the Water Framework Directive (Josefsson 2017). Therefore, the Water Framework Directive overrides the Urban Waste Water Directive if it provides more stringent objectives [see also Article 10(3) in the Water Framework Directive].

Similarly, in the Industrial Emissions Directive (2010/75/EU, Article 18) regulating for example production of pharmaceuticals, pesticides, and intensive rearing of poultry and pigs, there is a link to EQS: “*Where an environmental quality standard requires stricter conditions than those achievable by the use of the best available techniques, additional measures shall be included in the permit, without prejudice to other measures which may be taken to comply with environmental quality standards*”. Thus, based on the EQS values established for the environment, emission limit values could be set to reduce emissions from the main polluters, such as urban wastewater and intensive animal-rearing facilities. Similar implications (stricter pollution control) could be foreseen for any other polluting sources, such as fish farms and contaminated sites, in cases where significant emissions are causing an EQS exceedance in the receiving water.

## Discussion

Reducing risks for the emergence of antimicrobial resistance is complex and needs to be addressed using a combination of management measures in parallel addressing different parts of the lifecycle of antimicrobial substances. This study set out to establish if the Water Framework Directive could contribute to the much-needed risk reduction by including AMR as an aspect in the setting of EQS. From this analysis, we conclude that it is possible to take the indirect health risks from the environmental selection of AMR into account when deriving EQS for substances with antibiotic activity. This is based on a conclusion that human health concerns can be the main driver when setting an EQS that EQS can be based on additional data not specified in the guidance document and that there are no restrictions against establishing EQS using data on risks for AMR development. In addition, we see strong reasons to prioritise EQS on the EU level over national EQS, not the least because AMR travels across national borders. This, however, should not prevent individual member states to pave the way for EU-wide standards. We finally note that the Water Framework Directive can become a basis for setting emission levels related to risks for promoting AMR for municipal and industrial emissions within the EU, thereby taking precedence over other applicable legislation where such risks are not considered.

The objective of the proposal to include risks for selecting for AMR in the setting of EQS is to prevent or delay the emergence of new, successful types of resistance in pathogens, and with that associated excess morbidity, mortality, and societal costs. Establishing a direct, and ideally, quantitative link between antibiotic exposure concentrations in aquatic environments and the ultimate protection goals will be very difficult to establish. Partly, this is because of the complexity of the chain of events related to successful emergence that needs to align in time and space. But it is also related to the general difficulties in predicting the probabilities of rare events, all of which are in some way unique but share the feature that they have not yet occurred (Larsson and Flach 2022). This leaves us with basing risk management on known environmental risk factors for the emergence of resistance, where antibiotic pollution is the most accepted one (UNEP 2017; Access to Medicine Foundation 2021; European Commission 2019a; WHO 2020; O’Neil 2015).

Currently, there is no agreed-upon method for how to develop regulatory values such as EQS and PNECs protective against AMR, but there are several suggestions available in the literature (Larsson and Flach 2022), including approaches that make use of publicly available, standardised effect data (Bengtsson-Palme and Larsson 2016). Considering that the pharmaceutical industry itself has adopted one of these approaches as a basis for its voluntary emission targets (AMR Industry Alliance 2018), an agreement on what is good enough should be feasible. Still, to reach a wide acceptance, we would recommend that an expert committee is assigned by, for example,the European Commission to make a recommendation for how EQS that take into account resistance selection should be derived and maintained. These should be based on a method (or methods) that strikes a balance between scientific and regulatory considerations. For a new research field, the wish list for such a method may be difficult to completely check off. Nevertheless, it is desirable that the regulatory assessment method(s) applies to the aquatic environment that it can be applied for a variety of antimicrobial substances; that a rather simple assessment methodology is used; that it relies on publicly available data, in either peer-reviewed journals, open databases, or through the regulatory system; and that the most sensitive methods are used if all aspects are similar. The regulatory system also needs to be adaptive to change as this research field is developing rapidly.

As an example, demonstrating the possibility to move forward, ciprofloxacin was included as a river basin-specific pollutant in Swedish legislation in 2018 (then HVMFS 2013:19, now replaced by HVMFS 2019:25) (Swedish Agency for Marine and Water Management 2019). In the dossier, the EQS for resistance selection, based on MIC, and the conventionally derived EQS ended up at approximately the same level (0.1 μg/L), i.e. the EQS for resistance selection was not the driving value (Sahlin, Larsson, and Ågerstrand 2018). Nevertheless, the Swedish EQS for ciprofloxacin was implemented as a MAC-EQS (Maximum Allowed Concentration) rather than an AA-EQS (Annual Average), given the short generation time of bacteria. This is supported by the fact that resistance development can be a “one-time event” (Bengtsson-Palme and Larsson 2016; Larsson and Flach 2022). Had the MAC been based on conventional methodology, higher values could probably have been considered, given that the MAC-QS calculated in the dossier for the limnic and marine environments were 3.6 μg/L and 0.36 μg/L, respectively.

We are aware that there are additional risk scenarios related to AMR in the environment that are not captured by limiting risks for selection by antimicrobial residues. The transmission risks caused by direct discharges of already resistant bacteria from domestic animals and humans, particularly through wastewater treatment plants, also acquire legal attention. The bathing water directive (2006/7/EC) addresses risks associated with exposure to enteric pathogens, but it does not refer specifically to the risks with resistant bacteria. The very recent suggested revision of the Urban Waste Water Treatment Directive [European Commission 2022b)] proposes regular monitoring of AMR in both treated and untreated sewage in all WWTPs > 100,000 person equivalents in the EU, which may be a step in such a direction (Larsson et al. 2022).

## Conclusion and recommendation

In this paper, we have concluded that there are no formal obstacles in the Water Framework Directive or Environmental Quality Standards Directive to establish EQS values where the risk of selecting for AMR has been considered, although some clarifications/rewording in the legislation and the guidance document would likely promote this approach. As the Water Framework Directive will not be revised within the foreseeable future, the alternative could be to revise the EQS-Directive, especially the annexes but the simplest of these alternatives is to revise the CIS Guidance Document or establish a new one. Judging from the official documents from the EU, the environmental dimension of AMR does not seem to have been overlooked or considered insignificant. However, more actions are needed to manage this global concern. With this in mind, we recommend the following:

- The European Commission should establish an expert committee with the purpose to propose methods for deriving regulatory values like EQS and PNEC for resistance selection.
- The European Commission should ensure that the data needed for EQS derivation within the Water Framework Directive is provided by the market authorisation holder. Ranges of MIC values for different bacteria and/or other data to inform on a substance’s inherent ability to select for resistant genotypes (as well as data from ecotoxicity and environmental fate studies) should be added to the current information requirements. This is needed to make EQS derivations less dependent on publicly available data, which may be scarce, of insufficient quality, or not relevant for the assessment at hand.
- When developing EQS for antibiotic substances, the Member States and the European Commission should continue including EQS for resistance selection if data are available. This is important even in cases where the EQS for resistance selection is not the driving value since it provides a valuable comparison and contributes to method improvement.
